# The technical feasibility and preliminary results of minimally invasive endoscopic-TLIF based on electromagnetic navigation: a case series

**DOI:** 10.1186/s12893-021-01148-9

**Published:** 2021-03-20

**Authors:** Derong Xu, Shuo Han, Chao Wang, Kai Zhu, Chuanli Zhou, Xuexiao Ma

**Affiliations:** Department of Spine Surgery, The Affiliated Hospital of Qingdao University, Qingdao, 266000 Shandong China

**Keywords:** Electromagnetic navigation, Lumbar spondylolisthesis, Endo-TLIF, Percutaneous pedicle screw

## Abstract

**Background:**

Uniportal full endoscopic posterolateral transforaminal lumbar interbody fusion (Endo-TLIF) with percutaneous pedicle screw fixation is a promising, minimally invasive method for the treatment of lumbar spondylolisthesis. However, repeated radiation exposure from X-rays and the steep learning curve remain to be improved.

**Methods:**

This retrospective study explored the effects of electromagnetic navigation on improving Endo-TLIF with percutaneous pedicle screw fixation. Clinical information from 42 patients who had received Endo-TLIF with percutaneous pedicle screw fixation from May 2019 to November 2020 was analyzed retrospectively. The procedures were assisted under electromagnetic navigation. The rate of adjustment for guide wires, frequency of X-ray exposure, operative time, accuracy of pedicle screw location, and clinical outcomes were recorded.

**Results:**

The mean follow-up for 42 patients was 11.9 ± 3.1 months. The mean age of the patients was 56.1 ± 9.26 years, with a female/male ratio of 25:17. According to postoperative CT scans and 3D reconstructions, the excellent and good rate of pedicle screws was 96.4%. The rate of adjustment for guide wires under the assistance of electromagnetic navigation was 1.78%, and the frequency of X-ray exposure was 8.27 ± 1.83. The operative time was 167.25 ± 28.16 min, including the duration of guide wire insertion (14.63 ± 5.45 min) and duration of decompression and cage placement (75.43 ± 13.97 min). The duration of hospitalization after operation was 2.59 ± 1.16 days. The preoperative VAS score was 7.51 ± 1.91, and the preoperative ODI was 82.42 ± 8.7%. At the last follow-up, the VAS score was 2.09 ± 0.59, and the ODI was 11.09 ± 3.2%. There were statistically significant improvements in the VAS score and ODI in all patients at the follow-up (*p* < 0.05).

**Conclusions:**

Electromagnetic navigation can provide accurate positioning and guidance in real time, which improves the surgical efficiency of percutaneous pedicle screw placement and endoscopic decompression in Endo-TLIF with reduced radiation exposures.

**Supplementary Information:**

The online version contains supplementary material available at 10.1186/s12893-021-01148-9.

## Background

Lumbar spondylolisthesis is one of the most common causative factors of lower pain. It may reduce walking ability, affect quality of life, and cause lower limb pain when combined with instability and lateral recess stenosis. Traditional lumbar fusion with pedicle screws and interbody cage has been a popular treatment method; however, it requires extensive muscular decollement, laminectomy, and facetectomy, leading to massive blood loss, great damage to the normal spinal structure, epidural scar conglutination, and other complications [1, 2]. With the recent development of spinal endoscopy technology, minimally invasive surgery has found its application in the treatment of lumbar spondylolisthesis [3, 4]. Full endoscopic interlaminar ipsilateral and contralateral approach techniques have been applied to treat lumbar spondylolisthesis in the central, lateral recess, and extraforaminal regions [5]. Good clinical outcomes have been reported after endoscopic lumbar decompression with percutaneous pedicle screw fixation [6, 7].

The ideal position of the screws plays an important role in the success of uniportal full endoscopic transforaminal lumbar interbody fusion (Endo-TLIF) [8]. Guidance by fluoroscopy in lateral or anterior-to-posterior projection has been the most widely used method for percutaneous pedicle screw insertion. This approach depends on a surgeon’s individual skills and is accompanied by extended operative time, frequent intraoperative exposure to radiation, and a high rate of malposition [9]. In addition, the puzzling endoscopic anatomical structures are a great challenge for novices, which may prolong the operative time and increase intraoperative radiation exposure and the risk of bleeding and nerve injury [10]. Thus, seeking a technique to assist surgeons in locating and recognizing the endoscopic anatomy is required Additional files 1, 2, 3, 4, 5.

Navigation systems can provide precise positioning by device orientation relative to the patient's anatomy in real time [11]. They provide accurate positioning information for the patient's anatomy. Optical navigation and electromagnetic navigation are the most commonly used navigation systems in spinal surgery [12]. Most original navigation systems were based on optical tracking technology associated with infrared LEDs; patients’ preoperative image and anatomy could be integrated by relevant systems, so the movement of surgeons was tracked because the light from LEDs was captured by receiving cameras < Shurkhay > . The devices used were usually bulky and heavy, and the sight of LEDs should not be interrupted since it would disturb navigation. All of these may restrict the surgeon’s regular range of movement and result in poor handling [13].

The devices to be tracked in electromagnetic navigation are flexible because of the internal reference electrodes inside the instruments with minimized dimensions. The electromagnetic field penetrating the body eliminates the line-of-sight limitations of optical systems; thus, this approach is more appropriate for expanded applications in minimally invasive surgery and percutaneous procedures without disturbing normal operative workflow [14].

In this case series, an electromagnetic navigation system was used to assist pedicle screw insertion and endoscopic decompression in Endo-TLIF. The accuracy, safety, and efficiency were evaluated based on the analysis of pedicle screw position, radiation exposure, and operative time.

## Methods

Data for consecutive patients with single-segment lumbar spondylolisthesis who had received electromagnetic navigation–assisted Endo-TLIF with percutaneous pedicle screw fixation from May 2019 to November 2020 were retrospectively collected. These patients were selected because of persisting lower pain or claudication after at least 3 months of conservative treatment. Surgical indications were as follows: (1) single-segment grade 2 and below degenerative spondylolisthesis; (2) isthmic spondylolisthesis; (3) instability presentation in radiological examination; (4) neurogenic claudication because of unilateral lateral recess stenosis. Patients with spinal fusion surgery history, vertebral fracture, spinal tumor or infection, or spinal deformity with more than a 20-degree coronal curve were excluded. Clinical outcomes were assessed using the visual analog scale (VAS) and Oswestry Disability Index (ODI) scores for lower pain and leg pain at preoperative and follow-up examinations. Preoperative radiological studies included computed tomography (CT) scan with a 1 mm slice thickness and generated 3D reconstruction. The basic data, including age, gender, surgical segment, spondylolisthesis classification, and radiological image, were recorded. Our study was approved by the Affiliated Hospital of Qingdao University, and informed consent was signed by all patients.

### Electromagnetic navigation system

A new Virtu4D Guidance type of electromagnetic navigation system (Fiagon GmbH, Hennigsdorf, Germany) was adopted for our study. It is based on a new proprietary 4D-Vector field multi-array-sensor technology, which enables a smart, submillimetric precision in continuous tracking of the devices and the patient’s anatomical structures during the operation. With the smallest patient tracker in the market, smaller incisions and less visible scars can be achieved. Access Needle can bend during insertion into solid bony structures, and the Access Tracker adapts to the needle to assure continuous accuracy of guidance information. The system increases operative safety and efficiency by providing live and continuous three-dimensional imaging without disturbing or affecting normal surgical procedures.

### Surgical procedures of electromagnetic navigation–assisted Endo-TLIF

#### Preparation of electromagnetic navigation

We took L5/S1 segment as an example. All procedures were performed in the prone position under general anesthesia with the spine in slight flexion. Patients received transcranial electrical stimulation-induced motor-evoked potentials (MEPs) and electromyography (EMG) monitoring during the operation. The surgeon marked the bilateral pedicles of L5 and S1 roughly by C-arm positioning.

The field generator was fixed on the patient’s surface to generate the electromagnetic field. The devices fitted with signal coils inside were probed in the electromagnetic field. A reference coil (patient tracker) was fixed on the spinous process or the ilium to map the generated electromagnetic field with preoperative CT scan data. The devices used in the navigation system include a CenterPointer, Access Tracker, SpinePointer, and a matched navigable screwdriver. The CenterPointer was used for surface matching and pedicle drilling. The Access Tracker was used for entering into the vertebral body in a specified direction and for evaluating the screw length. The SpinePointer could detect the malpositions and destruction of the surrounding cortical bone. All instruments were interacted with the navigation system by a wire.

**Fig. 1:**
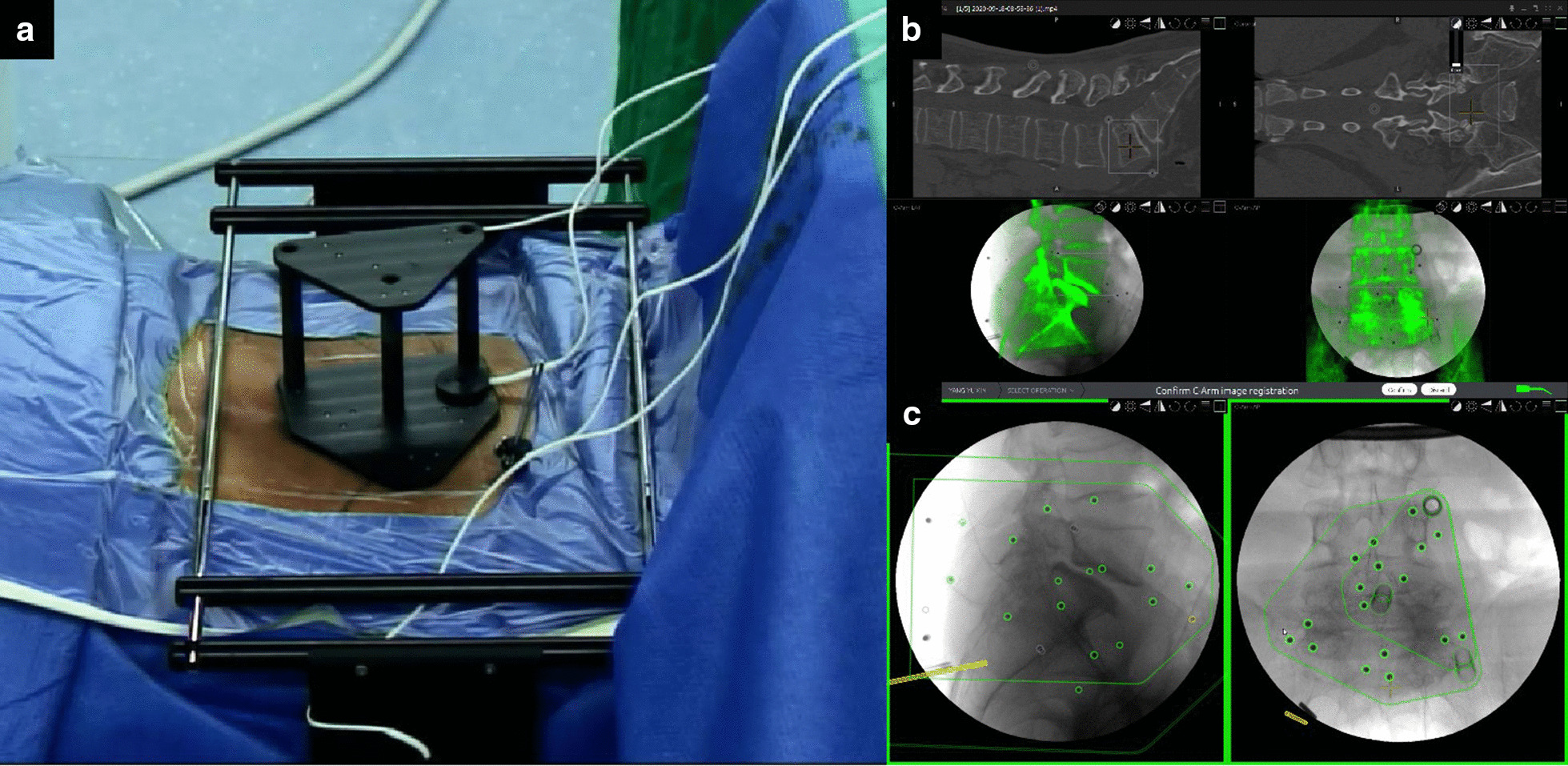
Preparation of electromagnetic navigation. **a** After the field generator and a patient tracker equipped with signal coils were fixed on the ilium, anteroposterior, and lateral fluoroscopic views of the lumbar segment were taken. **b**, **c** The software made surface matching on the respective vertebral body and compared the preoperative CT data in the electromagnetic coordinate system

After all instruments were in place, surface matching was performed with anteroposterior and lateral fluoroscopic views of the relevant segments and processed with the preoperative CT data set in the navigation system (Fig. 1)(Additional file 1). The surgeon performed the optical check with anatomical landmarks after systemic self-correction. It usually took 10–15 min to prepare the navigation system.

#### Guide wire insertion

**Fig. 2:**
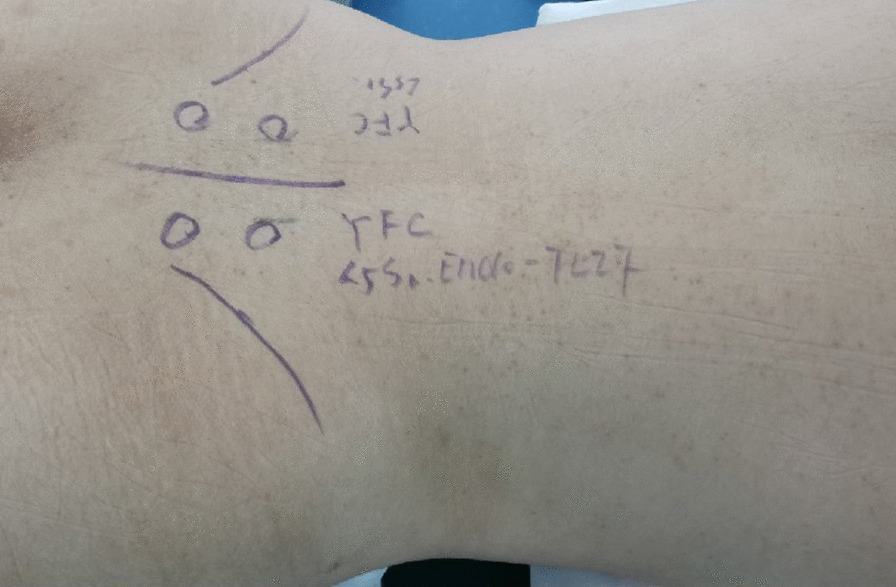
The incision design. According to C-arm positioning, bilateral iliac crest and pedicles were marked. In the electromagnetic navigation group, the incision was determined under the guidance of the Access Tracker, which could demonstrate the precise position of the trajectory to the pedicle. In the conventional group, the incision was roughly 5 cm to 6 cm away from the median spinous process

The incision was designed according to the precise position of the pedicles in the navigation system (Fig. 2). Under the guidance of Access Tracker, the surgical procedures in all spatial planes were visible in real time so that the surgeon could make dynamic regulation in a timely manner (Additional file 2). After drilling a pilot hole using Access Tracker, the surgeon continued to open through the pedicle and inserted a guide wire (300× 1.5 mm) under accuracy navigated guidance.

#### Decompression and cage placement under endoscope

**Fig. 3:**
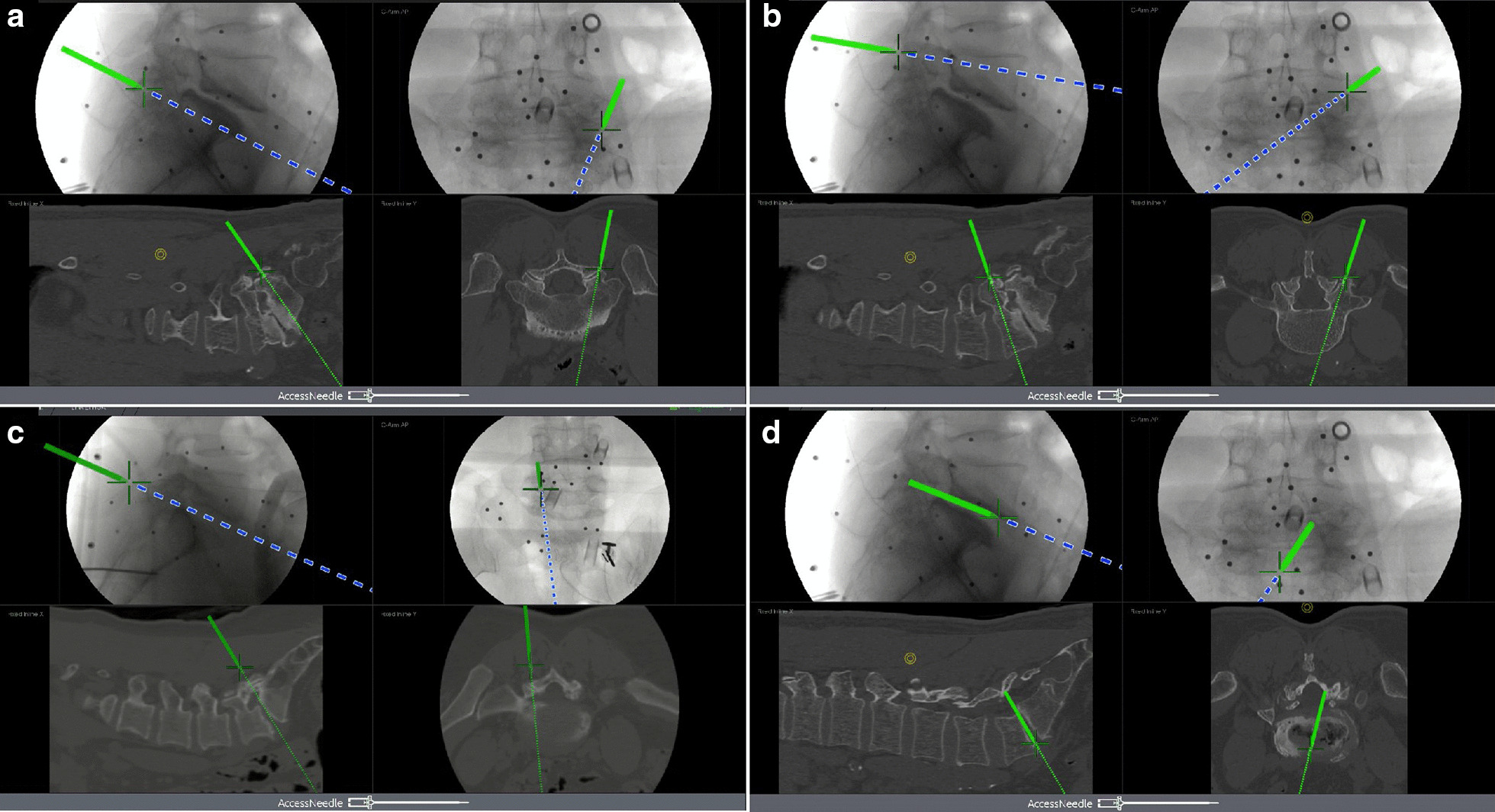
The application of electromagnetic navigation in multiple procedures. **a**, **b** Guide wire insertion. In the navigation system, the procedure and track of the Access Tracker were visible in all spatial planes in real time so the operator could make quick adjustment if needed. **c** After the uniportal endoscope was placed, the endoscopic anatomical structures such as S1 superior facet joint, L5 inferior facet joint tip, and isthmus could be recognized by Access Tracker in the navigation system. It also could assist with recognizing the location of the intervertebral disc for severe intervertebral collapse. **d** The Access Tracker evaluated the depth of processed intervertebral space

The endoscopic cannula was inserted on the undersurface of the superior facet using the Endo-Surgi PLUS endoscopic system (Joimax Inc.). After single-step tissue dilation and removing the expanding cannula, the uniportal endoscope was inserted. Under direct endoscopic visualization, the S1 superior facet joint, L5 inferior facet joint tip, isthmus, and part of the vertebral plate were shown and verified by Access Tracker in the navigation system (Fig. 3) (Additional file 3). A circular saw was used to drill a part of the S1 superior facet joint and L5 inferior facet joint tip to enlarge the foramen; ligamentum flavum, the dural sac, and S1 nerve root were exposed and protected by working cannula for spinal canal and bilateral contralateral lateral recess decompression. Continue to enlarge the foramen to the cephalic side and release the L5 nerve root.

During the operation, the surgeon was able to recognize the location and direction of the intervertebral disc with the assistance of Access Tracker in the electromagnetic navigation system (Fig. 3). The degenerated nuclear material was removed using reamers of different diameters through the working cannula. After an endplate preparation was finished, the Access Tracker was able to reach the end of the intervertebral space to evaluate the processed depth (Fig. 3).

The model cage was implanted to the center of the intervertebral space in appropriate depth, with the location confirmed by intraoperative anteroposterior and lateral fluoroscopic views. After removing the model and filling the intervertebral space with autograft and allograft, the cage was implanted via the working cannula to restore lumbar lordosis and the height of the intervertebral space. At last, the pedicle screws were installed to replace four guide wires, and the position of screws and cage were verified under C-arm fluoroscopy.

No drainage was placed, and all patients received postoperative dehydration, analgesia, anti-inflammatory, and neurotrophic treatment for 48 h.

The patients’ operation time, amount of bleeding, number of X-ray exposures, and hospitalization duration were recorded. After inserting the guide wires in the vertebral pedicle via electromagnetic navigation assistance or free-hand, C-arm fluoroscopy would prove whether their positions were optimal or not. We also evaluated the frequency of adjustment, referring to times of guide wires corrections before the pedicle screw could be placed satisfactorily. Postoperative CT scan with 3D reconstruction was performed to assess screw location by measuring the perpendicular distance between the pedicle cortical wall and the screw surface. The screw location was classified in four grades depending on the position of pedicle screws, as described by Neo et al. [15] (Table 1). Grade 0 and Grade 1 were defined as excellent and good position, respectively.

**Table 1:** Summary of computed tomography grading criteria

Grade	Accuracy of PPSP according to Neo et al. [15]
0	No deviation; the screw was contained in the pedicle
1	Deviation < 2 mm (i.e., less than half of the screw diameter)
2	Deviation > 2 and < 4 mm
3	Deviation > 4 mm (i.e., complete deviation)

We analyzed the data using SPSS version 18 statistical analysis software. Descriptive statistics were performed for all variables, and they were shown as mean and standard deviation (SD). Fisher’s exact test was used to analyze categorical variables. After verifying normal distribution, a paired *t*-test was used to compare preoperative and postoperative VAS scores and ODI scores. A value of P < 0.05 was considered significant.

## Results

We evaluated 42 patients followed up for 11.9 ± 3.1 months. Their mean age was 56.1 ± 9.26 years, and the female/male ratio was 25:17. Their information, including age, surgical segment, etiological classification, and Myerding classification, are presented in Table 2. There was no dural tear, nerve root injury, postoperative hematoma, postoperative infection, or other type of complication in this study.

**Table 2:** Basic information of the patients (n = 42)

	Electromagnetic navigation group
N	42
Age (years)	59.2 ± 10.15
Gender (Female/Male)	22/20
Surgical segment	
L3-L4	2
L4-L5	27
L5-S1	13
Etiological classification	
Isthmic	9
Degenerative	33
Meyerding classification	
I°	35
II°	7

According to postoperative CT scan and 3D reconstruction, the excellent and good rate of pedicle screws was 96.4% (Table 3). There were two patients presenting with Grade 3 misplacement of the pedicle screw, one of which had no clinical symptoms, and the other one suffered from lower extremity pain and numbness(Typical case 2). The rate of adjustment for guide wires under the assistance of electromagnetic navigation was 1.78%, and the frequency of X-ray exposure in the operation was 8.27 ± 1.83. The operative time was 167.25 ± 28.16 min, including the duration of guide wire insertion (14.63 ± 5.45 min) and the duration of decompression and cage placement (75.43 ± 13.97 min). The hospitalization time after operation was 2.59 ± 1.16 days.

**Table 3:** Pedicle grading of 42 patients

	Number of screws (n = 168)
Grade 0	140
Grade 1	22
Grade 2	4
Grade 3	2
Excellent and good rate (%)	96.4%

Grade 0 and Grade 1 was considered as excellent and good position (%)

The preoperative VAS score was 7.51 ± 1.91, and the preoperative ODI was 82.42 ± 8.7%. At the last follow-up, the VAS score was 2.09 ± 0.59, and the ODI was 11.09 ± 3.2%. There were statistically significant improvements in the VAS and ODI scores in all patients at follow-up (*p* < 0.05).

### Typical case 1

**Fig. 4:**
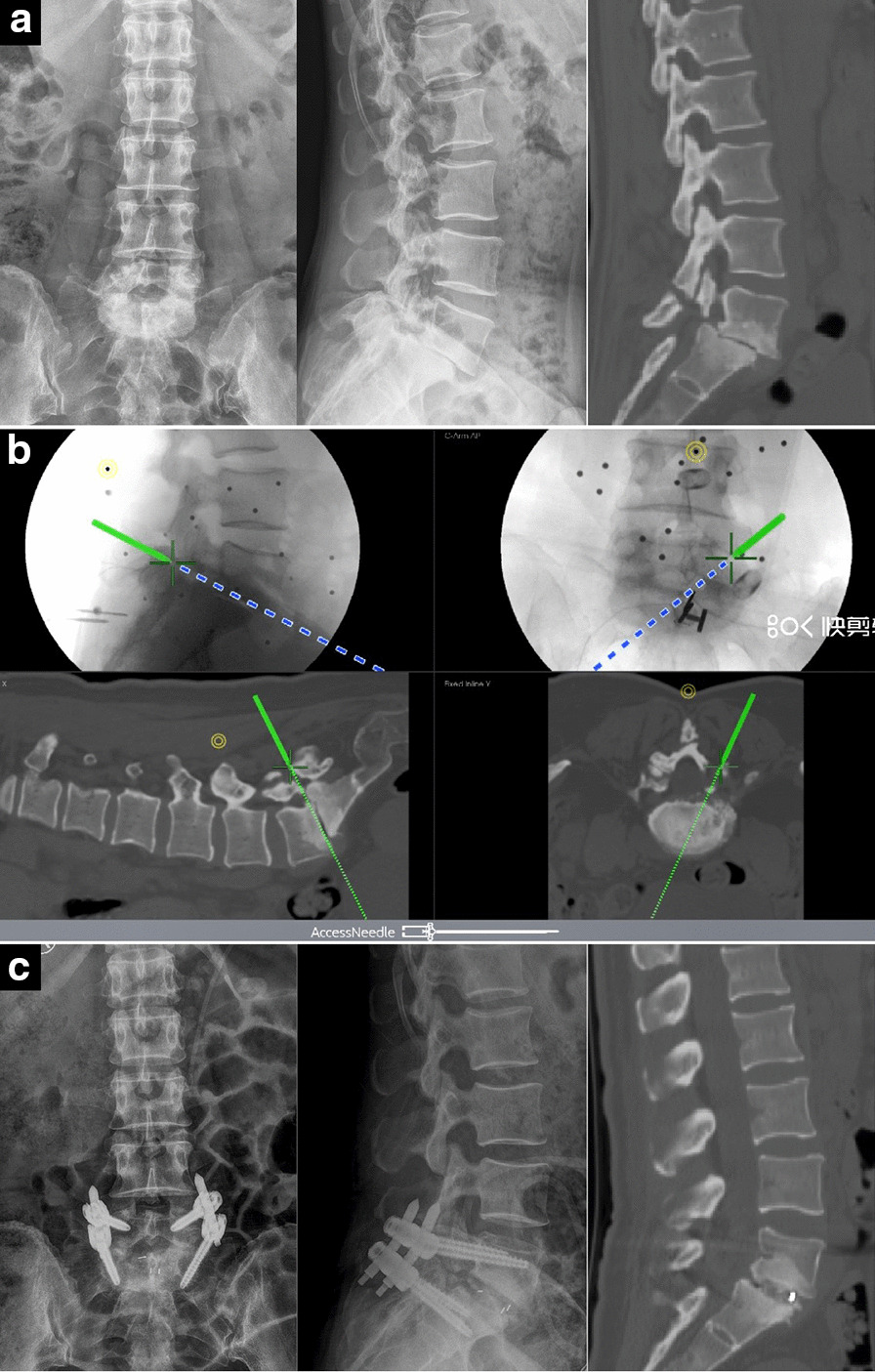
Images obtained in Case 1. **a** Preoperative images show grade 2 isthmic spondylolisthesis of L5; the intervertebral space almost disappeared, and there were huge osteophytes at the posterior margin of the vertebral body. **b** Electromagnetic navigation assisted with recognizing endoscopic facet joint and collapsed intervertebral space. **c** Postoperative images show that the reduction of spondylolisthesis and position of pedicle screws were perfect

A 48-year-old female suffered from lower pain for more than six years. The pain became worse in the previous month. On examination, "step sense" could be touched at L5/S1 level and her lumbar movement was obviously restricted. The preoperative radiographic examination revealed grade 2 isthmic spondylolisthesis of L5. The disappeared intervertebral space and presence of osteophytes at the posterior margin of the vertebral body could cause great difficulty in reduction and cage insertion. We took advantage of electromagnetic navigation to solve those problems successfully. Besides pedicle screw insertion and endoscopic cannula placement, under the assistance of electromagnetic navigation in recognizing endoscopic anatomical structure, it was easier to find the facet joint and collapsed intervertebral location even when there were obstructions caused by osteophytes. After removing the osteophytes by a specific bone chisel, the surgeon enlarged the intervertebral space by reamers and pedicle screw-rod distraction. The position of the cage was ideal under the direction of navigation. After operation, the patient achieved satisfactory reduction, and her clinical symptoms were obviously relieved (Fig. 4).

### Typical case 2

**Fig. 5:**
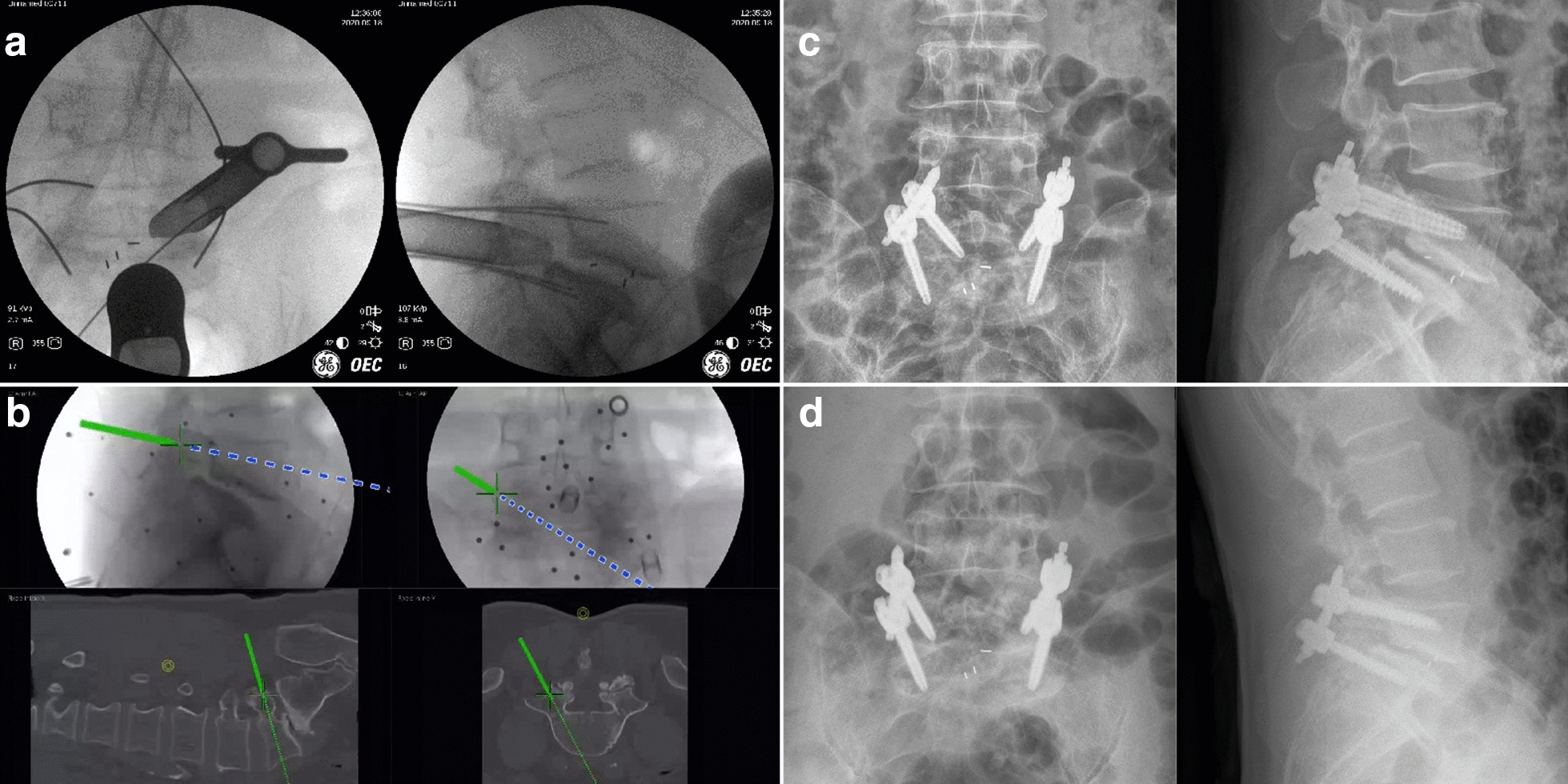
Images obtained in Case 2. **a** The X-ray fluoroscopy after cage insertion showed that the position of the left L5 pedicle guide wire was not satisfactory. **b** The surgeon adjusted the direction of the screws under the guidance of navigation again, ignoring the change in anatomical structure after slippage reset and intervertebral space restoration. **c** Postoperative X-ray fluoroscopy showed that the position of the left L5 pedicle screw was inward and upward, and the patient suffered from left lower extremity pain and numbness. **d** After a revision surgery to replace the left L5 pedicle screw, his left lower extremity symptoms were significantly relieved

A 53-year-old male patient diagnosed with isthmic spondylolisthesis of L5 received L5/S1 Endo-TLIF assisted by electromagnetic navigation. After the cage was inserted, the surgeon was dissatisfied with the position of the left L5 pedicle guide wire and adjusted the direction under the guidance of the navigation again. After the operation, the patient’s lower pain and right lower extremity symptoms were significantly relieved, but the left lower extremity pain and numbness aggravated, especially when walking. Postoperative examination showed that the position of the left L5 pedicle screw was inward and upward, so a revision surgery was performed to replace the left L5 pedicle screw. After the revision, the position of the left L5 pedicle screw was corrected and the symptoms of left lower limb were relieved (Fig. 5).

## Discussion

Compared to other kinds of surgeries for lumbar degenerative diseases, endoscopic minimally invasive treatments have been gaining increasing support from surgeons. Besides disc herniation and spinal stenosis, patients with instability and spondylolisthesis can also obtain better therapeutic effects through endoscopic fusion [16]. Several previous studies revealed positive clinical outcomes of the application of Endo-TLIF with percutaneous pedicle screw fixation. Wenjie Wu compared the application of Endo-TLIF with MIS-TLIF for the treatment of lumbar degenerative disease; they found that Endo-TLIF was effective for patients with single-segment lumbar degenerative diseases, and it was characterized by minor injury, quicker recovery, and lower cost [17]. Pang Hung Wu revealed that uniportal endoscopic posterolateral lumbar interbody fusion was feasible in the treatment of severe foraminal stenosis secondary to severe collapsed disc space, where the VAS score decreased 4.3 ± 1.0 at the last follow-up, and disc height was restored significantly [18]. In our study, the VAS score and ODI of the 42 patients decreased from 7.51 ± 1.91 and 82.42 ± 8.7% to 2.09 ± 0.59 and 11.09 ± 3.2%, respectively, and the clinical symptoms improved significantly at the last follow-up. The operation was accomplished through two incisions (2 cm to 2.5 cm in length, singly), and the duration of postoperative hospitalization was only 2 to 3 days. Endo-TLIF was shown to be as effective as open surgery, with less trauma and quicker recovery.

The manner of reducing the frequency of X-ray exposure and increasing the accuracy of percutaneous pedicle screws is an issue that warrants concern. The conventional percutaneous screw insertion techniques such as free-hand placement, fluoroscopy guidance, 3D fluoroscopy, and intraoperative CT/MRI are highly dependent on the operator’s experience and prone to errors regarding screw position [19]. In addition, repeated fluoroscopic use increases operative time and increases physical harm to operators and patients from radiation exposure. With the guidance of navigation techniques, operator could achieve higher accuracy during screw insertion compared with free-hand or fluoroscopy techniques. Hahn studied the application of an electromagnetic navigation system for pedicle screw insertion in the lumbar spine of human cadavers and showed an excellent chance of success, compared to other techniques [14]. In our study, the results showed a higher accuracy of first guide wire insertion and less adjustment for the screw canal in the electromagnetic navigation group than which has been reported for conventional techniques. The postoperative CT scan showed the excellent and good rate of pedicle screws in 96.4%, with decreased operative time and radiation exposure.

A minimally invasive endoscopic decompression is a complicated transforaminal approach, which has a steep learning curve. In some particular cases, such as intervertebral collapse, severe hyperosteogeny, and revision surgery, even experienced surgeons may be troubled by complicated anatomical structures under the endoscope [20]. This challenge limits broader adoption of the procedure. The electromagnetic navigation system provides clarity to this procedure and enables guidance with a small single device, the Access Tracker. Along with the small IseeTracker, a noninvasive automatic registration enables all the benefits of this minimally invasive surgical procedure, and the Access Tracker could show their positional relationship with the surrounding structures, as well as a quicker learning curve. During decompression, the Access Tracker could further assist with recognizing superior facet joint, inferior facet joint tip, isthmus, and part of the vertebral plate. Before removing the intervertebral disc tissues, the operator could use the Access Tracker to detect a severe collapsed disc (Additional file 4). Finally, the Access Tracker could evaluate the depth of processed intervertebral space before cage insertion (Additional file 5). All of these improved the safety and precision of the operation.

In this study, there were two patients with Grade 3 misplacement of the pedicle screw. One of them suffered from lower extremity pain and numbness. These symptoms were caused by the adjustment of the guide wire after cage insertion, which was under the guidance of electromagnetic navigation without re-registration. That case emphasizes that when performing percutaneous screw placement assisted by electromagnetic navigation, after the cage placement, slippage reset and the height of the intervertebral space would be restored, so that electromagnetic navigation would be invalid because the preoperative CT data no longer match the present anatomical structure (Fig. 5). Re-registration was required to match the present spine structure with electromagnetic navigation in a timely manner.

In the course of our application, we found that there were still some technical defects with electromagnetic navigation. The electromagnetic navigation may be disturbed by the C-arm fluoroscope or other kinds of metal devices after image matching. It takes a long time to complete the registration because of the complicated procedures, which is difficult for novices. The system should be more convenient and efficient during the operation, with further simplified registration and matching and improved anti-interference capability and self-correcting for errors. Further application of electromagnetic navigation for cervicothoracic junction or other special regions should be developed.

This was a clinical study intended to evaluate a new electromagnetic navigation system for positioning pedicle screws and endoscopic decompression in the lumbar Endo-TLIF surgery. Some limitations could not be ignored in our present study. First, this was a retrospective study to evaluate patients performed Endo-TLIF, therefore, inherent differences and selection bias of the enrolled cases was inevitable. Second, the sample size was relatively small and the follow-up time was not long enough, so it failed to fully evaluate the long-term clinical outcomes and complications. In the future, we need a prospective, randomized controlled trial in multiple centers with a large sample size and longer follow-up to eliminate these biases.

## Conclusion

In our study, we demonstrated the technical feasibility and preliminary results of Endoscopic-TLIF based on electromagnetic navigation. It is proved that electromagnetic navigation assisted Endo-TLIF is an effective minimally invasive surgical management technique with higher accuracy and reduced radiation exposures for lumbar spondylolisthesis.

## Supplementary Information

**Additional file 1.** The roughly surface matching according to fluoroscopic views and preoperative CT data set in the navigation system.

**Additional file 2.** The Access Tracker assisted to pedicle screw insertion.

**Additional file 3.** The navigation system could verify the endoscopic position and direction.

**Additional file 4.** The Access Tracker assisted to detect a severe collapsed disc.

**Additional file 5.** The Access Tracker could evaluate the depth of processed intervertebral space before cage insertion.

## Data Availability

The datasets generated and analyzed during the current study are available from the corresponding author on reasonable request.

## Abbreviations

Endo-TLIF: Uniportal full endoscopic transforaminal lumbar interbody fusion
VAS: Visual analog scale
ODI: Oswestry Disability Index
MEPs: Motor-evoked potentials
EMG: Electromyography
