# Supplementary figures and images for: The technical feasibility and preliminary results of minimally invasive endoscopic-TLIF based on electromagnetic navigation: a case series
Source: BMC Surg. 2021 Mar 20;21:149. doi: 10.1186/s12893-021-01148-9 (PMC7981827; doi:10.1186/s12893-021-01148-9)

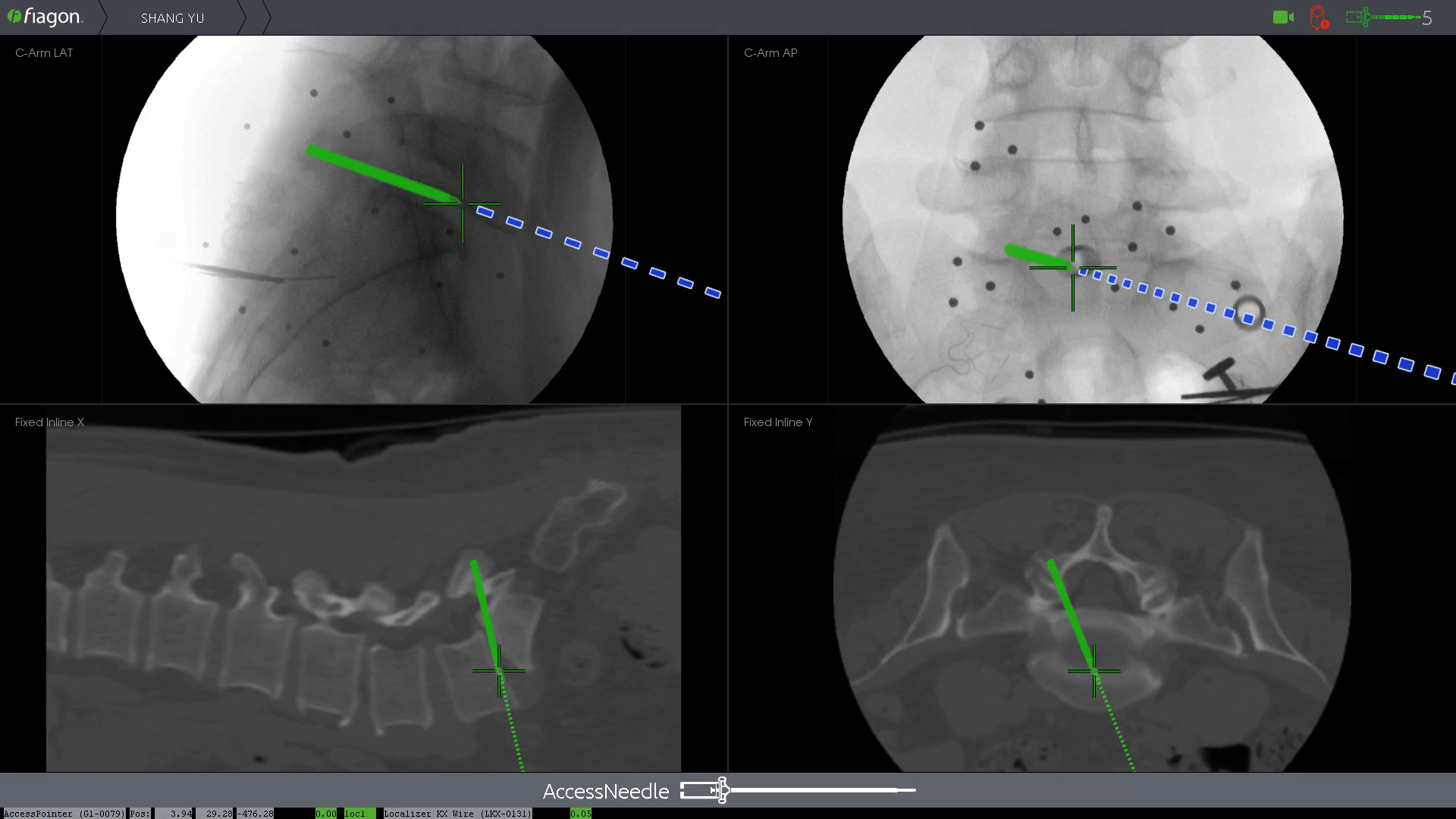

Supplement: Supplementary file 1 — Additional file 1. The roughly surface matching according to fluoroscopic views and preoperative CT data set in the navigation system. [file 12893_2021_1148_MOESM1_ESM.gif]

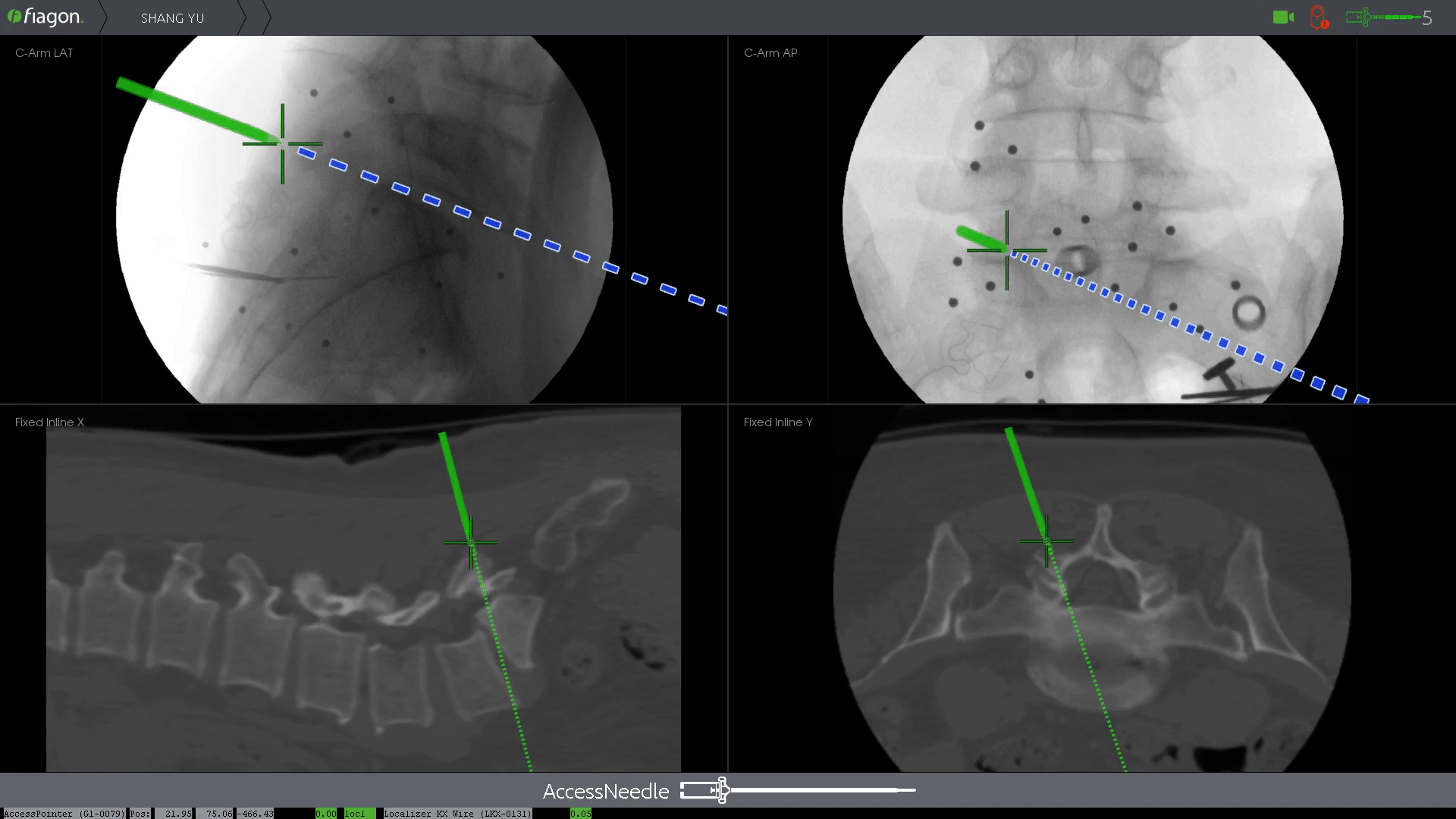

Supplement: Supplementary file 2 — Additional file 2. The Access Tracker assisted to pedicle screw insertion. [file 12893_2021_1148_MOESM2_ESM.gif]

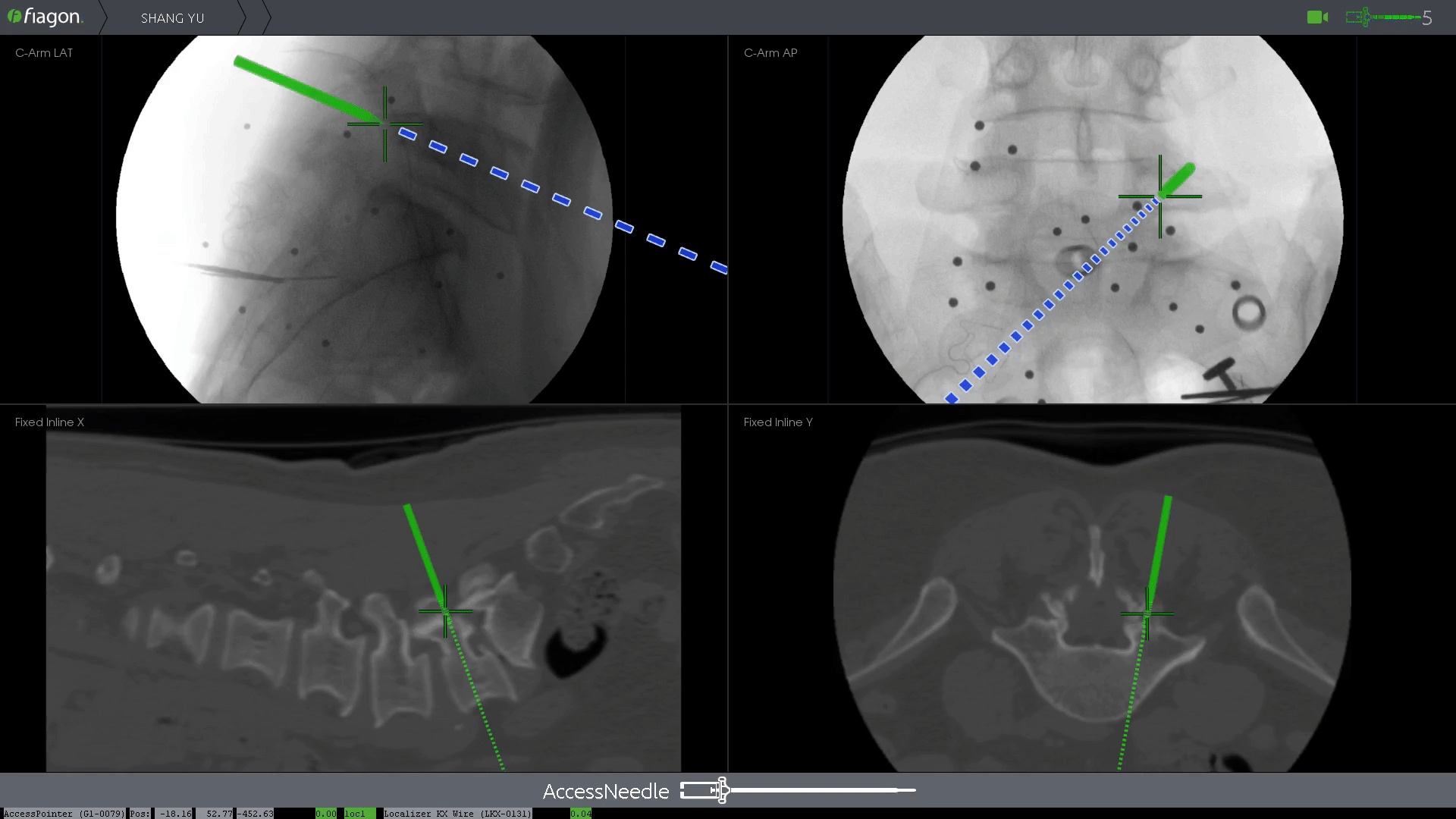

Supplement: Supplementary file 3 — Additional file 3. The navigation system could verify the endoscopic position and direction. [file 12893_2021_1148_MOESM3_ESM.gif]

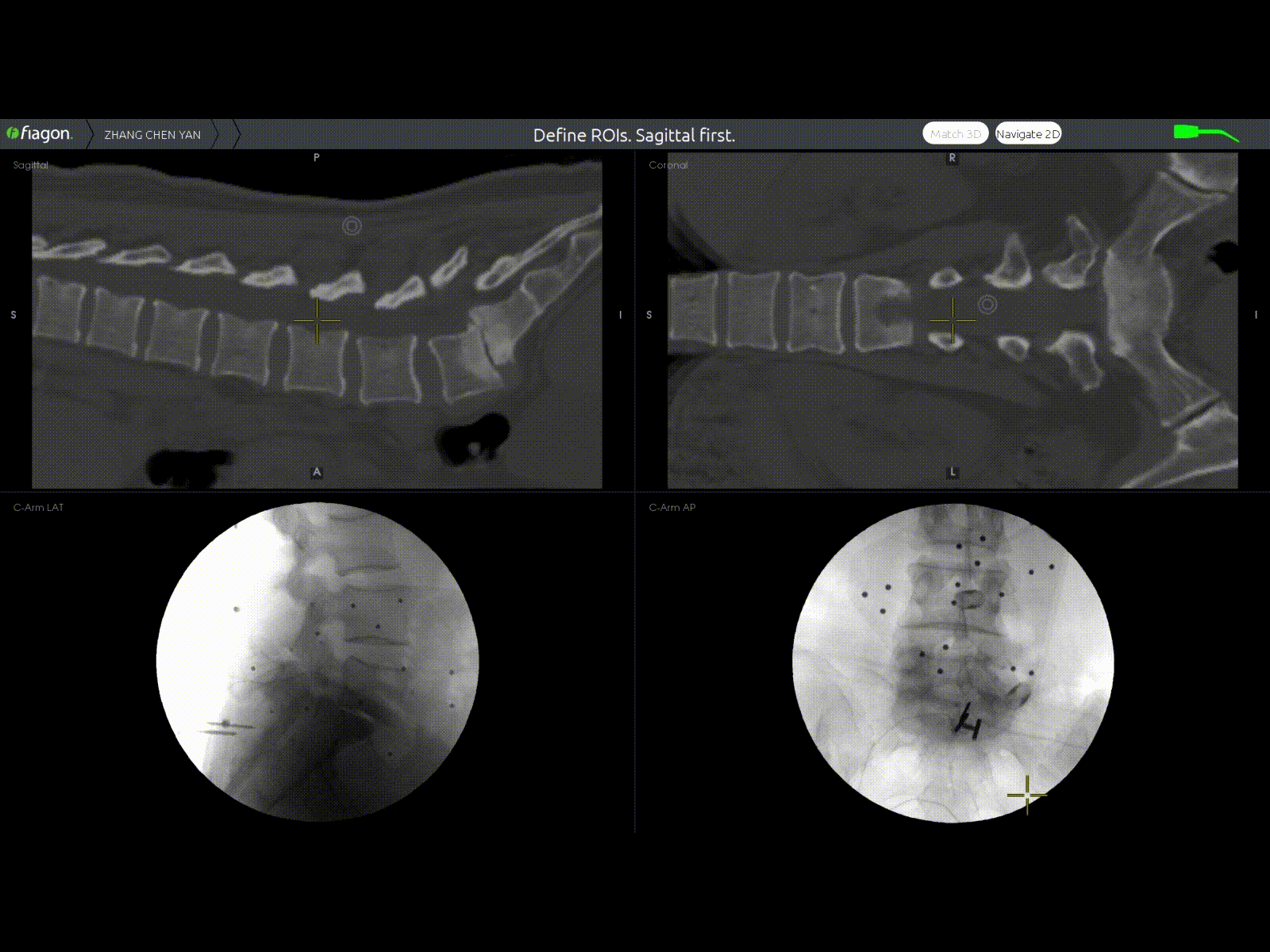

Supplement: Supplementary file 4 — Additional file 4. The Access Tracker assisted to detect a severe collapsed disc. [file 12893_2021_1148_MOESM4_ESM.gif]

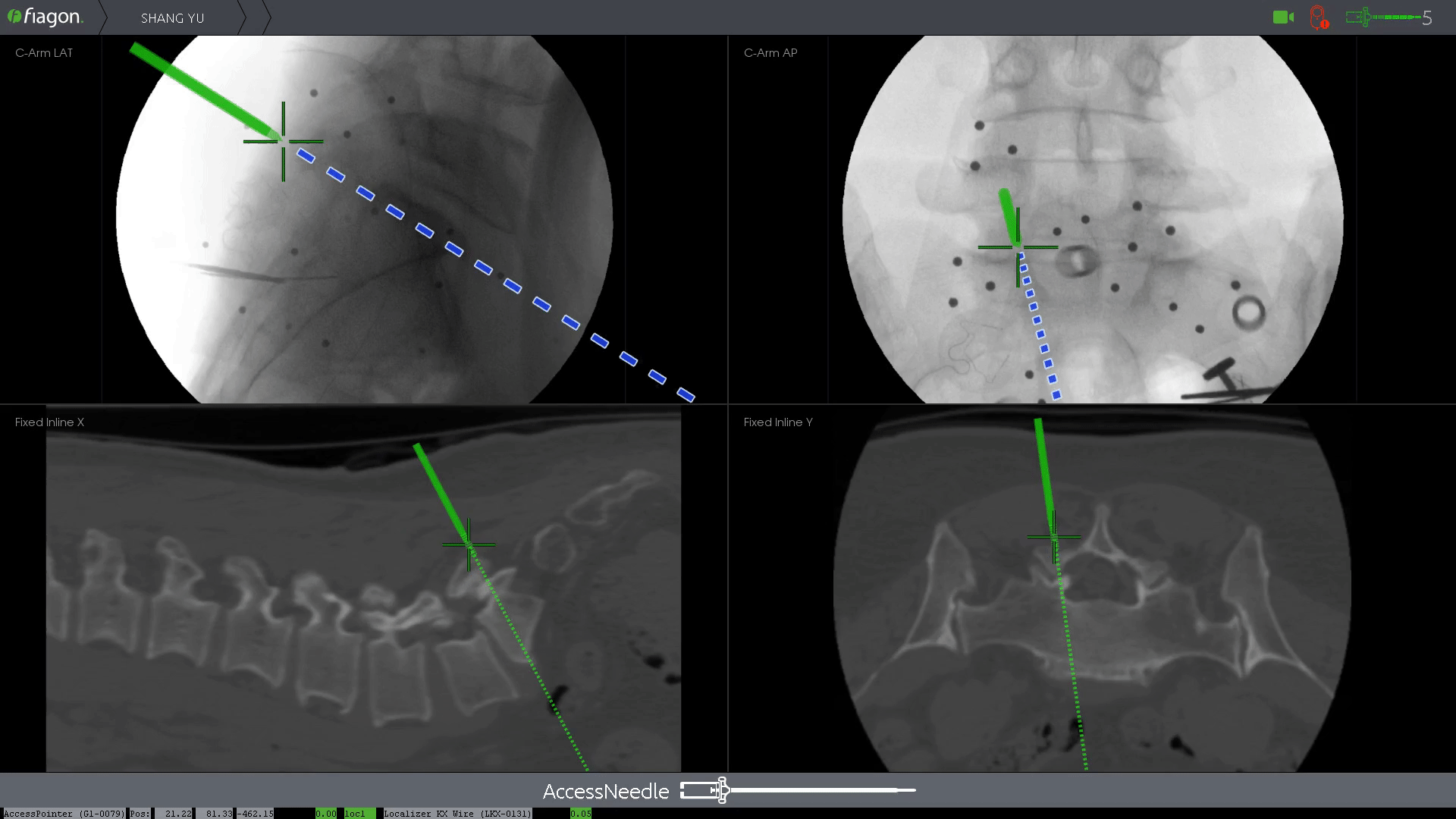

Supplement: Supplementary file 5 — Additional file 5. The Access Tracker could evaluate the depth of processed intervertebral space before cage insertion. [file 12893_2021_1148_MOESM5_ESM.gif]
